# Endemism and Conservation of Hemiptera on the Qinghai‐Tibetan Plateau

**DOI:** 10.1002/ece3.73244

**Published:** 2026-03-19

**Authors:** Zhengxue Zhao, Xueli Feng, Jing Zhou, Xiudong Huang

**Affiliations:** ^1^ College of Agriculture Anshun University Anshun China; ^2^ Guizhou Technological College of Ecology and Energy Guiyang China; ^3^ Anshun Academy of Agricultural Sciences Anshun China

**Keywords:** conservation, endemism hotspot, Hemiptera, historical climate change, Qinghai‐Tibetan plateau

## Abstract

The Qinghai‐Tibetan Plateau is essential for the maintenance of global biodiversity. Exploring the drivers of species diversity and the conservation status of species in this region contributes significantly to elucidating the mechanisms underlying diversity patterns and optimizing conservation strategies. Understanding endemism is key to developing effective conservation strategies; yet, it has not been fully investigated in insects on the Qinghai‐Tibetan Plateau. To address this gap, we compiled a species distribution dataset for Hemiptera and analyzed the relationships between weighted endemism (WE) patterns and six categories of environmental variables using statistical models. We also identified endemism hotspots based on the top 10% grids with the highest WE values and overlapped them with protected areas to investigate the effectiveness of conservation. Our results revealed that endemism patterns on the Qinghai‐Tibetan Plateau are heterogeneously distributed and are mostly determined by historical climate change. Furthermore, we identified three endemism hotspots located in the eastern, southeastern, and southwestern fringes of the plateau and defined them as high‐priority conservation areas. We revealed that the first two of them are inadequately safeguarded by protected areas, thereby showing a low level of conservation performance for endemism. Based on our findings, we recommend incorporating endemism into research efforts to elucidate the mechanisms shaping species diversity patterns on the Qinghai‐Tibetan Plateau, as it provides important historical information on the processes driving these patterns. Moreover, it is necessary to optimize the current protected area network via the expansion of existing protected areas or the establishment of new protected areas.

## Graphical abstract

Endemism patterns of Hemiptera on the Qinghai‐Tibetan Plateau (QTP) are mainly driven by historical climate change. Existing protected areas do not sufficiently cover endemism hotspots.

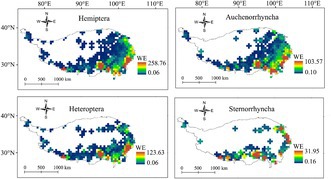

## 1 Introduction

Many regions of the world exhibit extreme levels of species diversity and endemism and are thus priority targets for conservation (Myers et al. 2000; Marchese 2015). This conservation need has prompted extensive research into the spatial patterns of biodiversity and the driving mechanisms underlying these patterns (e.g., Huang et al. 2008; Feng et al. 2016, 2023). The Qinghai‐Tibetan Plateau (QTP), known as the “Roof of the World,” is located in southwestern China and is the highest and largest plateau (with an area of approximately 2.5 × 10^6^ km^2^) in the world. Four out of 36 global biodiversity hotspots are located on the QTP; namely, the Mountains of Central Asia, the Himalaya, the Indo‐Burma ranges, and the Mountains of southern‐central China. The region's diverse environmental conditions, complex topography, and unique geological history combined to create and maintain remarkable biodiversity (Favre et al. 2015; Zhang et al. 2017) with a high degree of endemism (Yu et al. 2019; Deng et al. 2020). However, the QTP is highly sensitive to environmental change (Liu and Chen 2000), including habitat loss driven by rising temperatures and increased human activities (Liu et al. 2021; Mao et al. 2023; Yang et al. 2025), culminating in a significant decline in biodiversity. Thus, biodiversity conservation currently faces huge challenges under the pressure of these rapidly changing environmental conditions.

The findings from studies on the main factors driving species diversity patterns across different taxa were inconsistent in a given region (Hawkins et al. 2003; Qian 2010), indicating that the mechanisms underlying species diversity patterns differ. Similar results were documented on the QTP. For example, modern climate was shown to be the primary driver of plant diversity patterns (Yan et al. 2013; Zhang et al. 2021; Li et al. 2022), whereas topographical heterogeneity and temperature amplitude were shown to jointly regulate the processes shaping bird diversity patterns (Zhang et al. 2017). These findings suggest that cross‐taxon studies are urgently needed to understand the mechanisms of species diversity patterns on the QTP. To our knowledge, there remain very few such studies on insects.

Hemiptera is an ancient order of insects that can be traced back to the Late Devonian‐Early Carboniferous (Misof et al. 2014; Johnson et al. 2018). Currently, more than 100,000 hemipteran species have been described worldwide, making it the fourth largest insect order (Song et al. 2024). Most species in this order are herbivorous, such as aphids, scale insects, and leafhoppers, and only a small number are predatory. Hemipteran species are widely distributed in diverse environments, due to their small size, rapid life cycle, and quick reproduction rate (Schuh and Slater 1995; Forero 2008). These characteristics make them the frequent subjects of biogeographical research (Diniz‐Filho et al. 2013; Wei et al. 2016; Pinedo‐Escatel et al. 2021; Roell et al. 2025). The diversity patterns of Hemiptera (including specific lineages within the order) and their underlying mechanisms have been investigated in different regions all over the world. Studied families include, for example, the Pentatomidae (Ferrari et al. 2010; Poester‐Carvalho et al. 2023) and the Triatominae (Heteroptera: Reduviidae) (Diniz‐Filho et al. 2013; Ferrari et al. 2022). A recent study considering multiple variables revealed that topographic heterogeneity largely influences the heterogeneous distribution of the Hemiptera insect group (Li et al. 2019). Unfortunately, this study did not explore the relationship between endemism patterns and environmental variables. Endemism refers to the biogeographic phenomenon in which a species is restricted to a specific or limited geographical area (Anderson 1994; Guerin et al. 2015). Some early studies revealed that endemism patterns are not only strongly influenced by topographic heterogeneity, but also closely related to historical climate change (Schuldt and Assmann 2009; Zhao et al. 2020). It is currently uncertain whether the key factors driving Hemiptera endemism patterns are consistent with those driving species richness patterns on the QTP. It is important to note that most insects have a high degree of endemism, which suggests that they have low mobility and therefore a lower probability of postglacial recolonization (Zhao et al. 2020). As a result, historical climate change plays a larger role compared to other environmental variables. Thus, the inability to identify the key drivers of endemism patterns hinders a deeper understanding of the mechanisms underlying species diversity patterns.

Owing to its exceptional biodiversity and high sensitivity to climate change, the QTP has become a key region for global biodiversity conservation. Identifying biodiversity hotspots is a crucial step in conservation efforts, as it allows for the maximization of species protection with limited resources. In this process, the protection of endemic species is prioritized, considering that they have a high extinction rate (Ohlemüller et al. 2008; Rønsted et al. 2022). Biodiversity hotspots merely indicate which regions are worthy of priority conservation; however, effective conservation still depends on the establishment of protected areas. The Chinese government has established numerous protected areas on the QTP to cope with biodiversity loss. Nevertheless, recent extensive studies have shown that the existing protected areas in this region do not fully cover all plant and vertebrate animal hotspots (Yu et al. 2021; Liang et al. 2021; Zhang et al. 2024), suggesting that the protected areas are incomplete and in urgent need of improvement. At present, the effectiveness of protected areas in safeguarding insect species has rarely been evaluated. Therefore, using Hemiptera data to assess existing gaps in the protection of biodiversity hotspots can greatly benefit the design of future protected areas and consequently improve conservation effectiveness.

Based on the background information presented above, in this study, we compiled a comprehensive species distribution dataset for Hemiptera on the QTP to (1) determine the most critical factors driving endemism patterns; (2) identify endemism hotspots to establish conservation priority areas; and (3) assess whether existing protected areas sufficiently cover endemism hotspots and thus determine conservation effectiveness. We hypothesize that historical climate change is more closely associated with endemism distribution patterns and that current protected areas are not effectively safeguarding Hemiptera endemism.

## 2 Materials and Methods

### 2.1 Species Distribution Dataset

**FIGURE 1:**
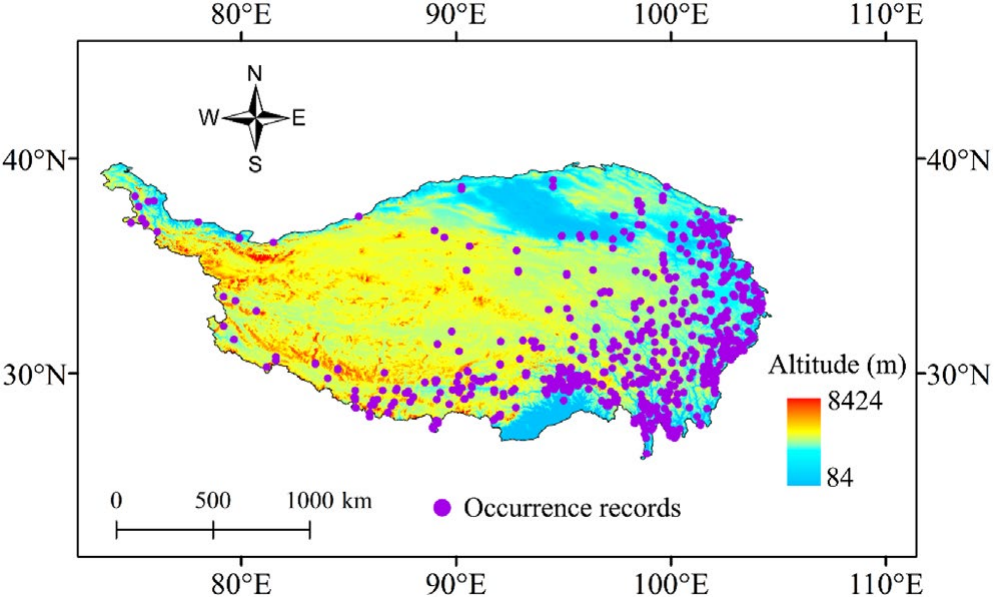
Map showing the 3502 occurrence records for Hemiptera on the Qinghai‐Tibetan Plateau (QTP). Purple dots represent the locations of species occurrences.

Raw occurrence records for Hemiptera on the QTP were collected from the following three sources: (1) published literature, (2) specimen records from Guizhou University, and (3) the Global Biodiversity Information Facility (GBIF, https://www.gbif.org/), accessed on August 15, 2025. These sources represent a collection of a wide range of occurrence records to reduce sampling bias related to the reliance on a single collection source. The accuracy and validity of the occurrence records from the first two data sources were carefully verified, and any existing geographic coordinates were checked via Google Earth. Moreover, occurrence records assigned at a spatial resolution at the county level or finer were used, and latitudes and longitudes were identified using Google Earth if they were missing. When processing the GBIF‐derived occurrence records, we first eliminated records with common collection errors (e.g., sea coordinates and zero coordinates) and then excluded records with a spatial uncertainty ≥ 50 km via the CoordinateCleaner package in R 4.3.3. Finally, we obtained a species distribution dataset consisting of 3502 occurrence records for 1542 species (Figure 1; Table S1).

### 2.2 Endemism Patterns

To obtain endemism patterns, weighted endemism (WE) values were calculated for 0.5° × 0.5° grid cells (about 50 km × 50 km) by counting the sum of the inverse of the number of grids occupied by each species via the raster and terra packages in R 4.3.3 (Equation 1). A grid size of 0.5° was selected since the spatial resolution of most of the occurrence records was less than 50 km. This step minimizes the amount of occurrence records assigned to incorrect grid cells, but still retains the fine spatial resolution to depict endemism patterns. Moreover, the selected grid size is in accordance with a previous study of Hemiptera richness patterns on the QTP, which facilitates comparison of results (Li et al. 2019). The WE index applies a simple continuous weighting function to retain the conceptual continuity of endemism (Laffan and Crisp 2003). Species with a narrow distribution range have higher weights, whereas those with a wide range are assigned lower weights. Additionally, to reduce the influence of sampling bias on endemism patterns, a moving window analysis with circle type and a radius of 0.5 was employed (González‐Orozco et al. 2011). WE values were calculated not only for Hemiptera as a whole but also for three suborders for which species distribution data were available: Auchenorrhyncha, Heteroptera, and Sternorrhyncha.

(1) $$\text{WE}=\sum_{i=1}^{n}\frac{1}{R_{i}}$$

where *n* is the number of species appearing in a 0.5° grid cell and *R*_*i*_ is the total number of 0.5° grid cells occupied by a species.

### 2.3 Environmental Variables

This study investigated the relationships between WE patterns and 15 environmental variables grouped into the following six categories: (1) temperature: annual mean temperature (MAT), max temperature of warmest month (MTWM), and mean temperature of the warmest quarter (MTWQ); (2) precipitation: annual precipitation (AP) and precipitation of the warmest quarter (PWQ); (3) climate seasonality: temperature annual range (TAR), temperature seasonality (TS), and precipitation seasonality (PS); (4) historical climate change: change in MAT since the last glacial maximum (MATC) and change in AP since the last glacial maximum (APC); (5) topographic heterogeneity: elevation range (ER), slope (SP), and terrain ruggedness index (TRI); (6) vegetation: normalized difference vegetation index (NDVI) and enhanced vegetation index (EVI). The six categories of variables were selected because they contribute to the richness patterns of Hemiptera on the QTP (Li et al. 2019) and Hemiptera are predominantly herbivorous. To align with WE values in the 0.5° × 0.5° grids, grids with the same cell size and extent were created using ArcGIS 10.7 and then the values for temperature, precipitation, climate seasonality, vegetation, SP, and TRI in each 0.5° grid were represented by the average value of all pixels in that grid. MATC and APC were calculated as the absolute value of the difference between current MAT/AP and MAT/AP in the last glacial maximum. ER is the difference between the maximum and minimum elevations in the 0.5° grid.

NDVI and EVI raster layers with a spatial resolution of 1 km were downloaded from the Resource and Environmental Science Data Platform (https://www.resdc.cn/). The raster layers of temperature, precipitation, climate seasonality, and elevation were obtained from the WorldClim database (https://www.worldclim.org) at a spatial resolution of 30 s, except for MAT and AP in the last glacial maximum, which were obtained at a spatial resolution of 2.5 min from mean values derived from the CCSM4, MIROC‐ESM, and MPI‐ESM‐P climate models. The raster layers of SP and TRI were created by the terra packages in R 4.3.3 with an elevation raster.

### 2.4 Statistical Analysis

Before analysis, WE values were log10‐transformed and then they and all environmental variables were standardized (mean = 0 and standard deviation = 1). The relationships between each environmental variable and WE were assessed using the ordinary least squares (OLS) model in Hemiptera and three suborders. The issue of spatial autocorrelation potentially increasing the significance of the model was addressed by assessing the correlation between observed and estimated WE values (Clifford et al. 1989; Qian 2008). Non‐significant variables were excluded from subsequent analyses. Moreover, to determine the relative importance of the categories of environmental variables, multiple OLS models were constructed using one variable from each category. All variables within each category were considered, and the model with the lowest Akaike Information Criterion Corrected value was selected as the optimal model. We checked that the variance inflation factor values of the environmental variables included in the optimal model were < 5, which indicated the model had low collinearity. Next, the model was subjected to hierarchical partitioning, and the relative importance of each environmental variable was represented using independent effect. All statistical models were conducted using the car, glmm.hp., stats, AICcmodavg, and SpatialPack packages in R 4.3.3.

### 2.5 Identification of Endemism Hotspots and Assessment of Conservation Effects

The top 10% grids with the highest WE values were considered as the threshold to define endemism hotspots in ArcGIS 10.7. This threshold has been extensively used in previous studies (Noroozi et al. 2019; Zhao et al. 2023) and successfully implemented in the QTP (Li et al. 2024). Furthermore, the maps of protected areas were overlaid with the endemism hotspot grids to explore conservation effectiveness within them. An endemism hotspot grid was regarded as protected if more than 30% of its area was under protection. This is in line with the 30% protection target established for 2030. Conversely, if less than 30% of the area was under protection, the grid was considered not protected. The maps of protected areas were obtained from the National Earth System Science Data Center (http://www.geodata.cn/).

## 3 Results

**FIGURE 2:**
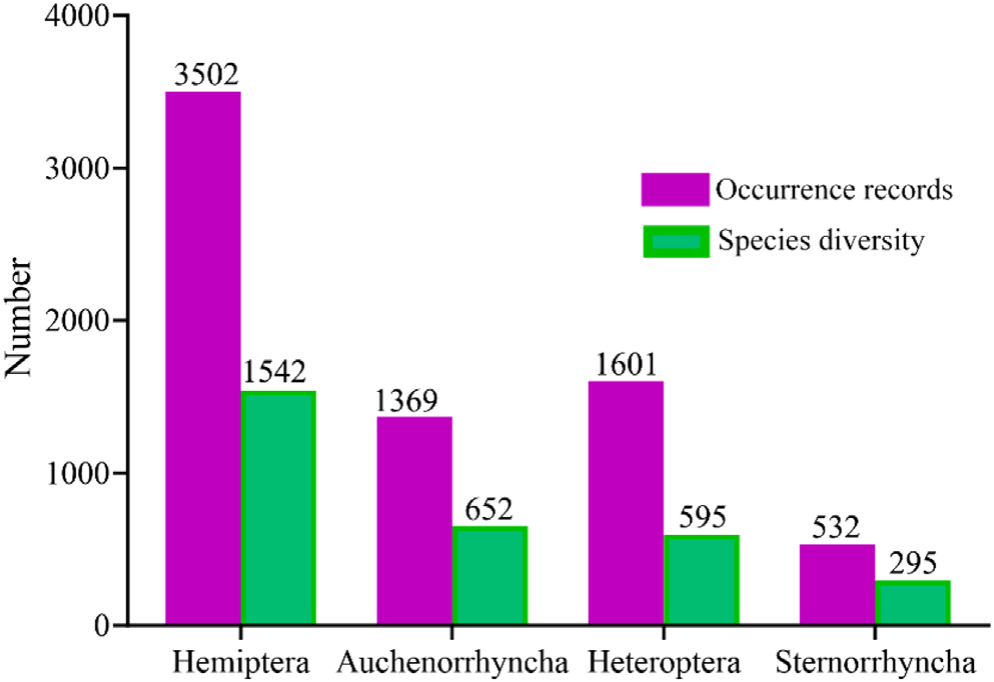
Number of species and occurrence records of Hemiptera (overall+three suborders) on the Qinghai–Tibetan Plateau (QTP). Purple bars indicate the number of occurrence records, and green bars indicate the number of species.

Among the three Hemiptera suborders examined, Auchenorrhyncha had the highest species richness (42.28% of the total) and ranked second in terms of the number of occurrence records (39.09%) (Figure 2). Heteroptera ranked second in terms of species richness (38.58%) but had the highest number of occurrence records (45.71%) (Figure 2), whereas Sternorrhyncha had the lowest species richness (19.13%) and number of occurrence records (15.19%) (Figure 2).

**FIGURE 3:**
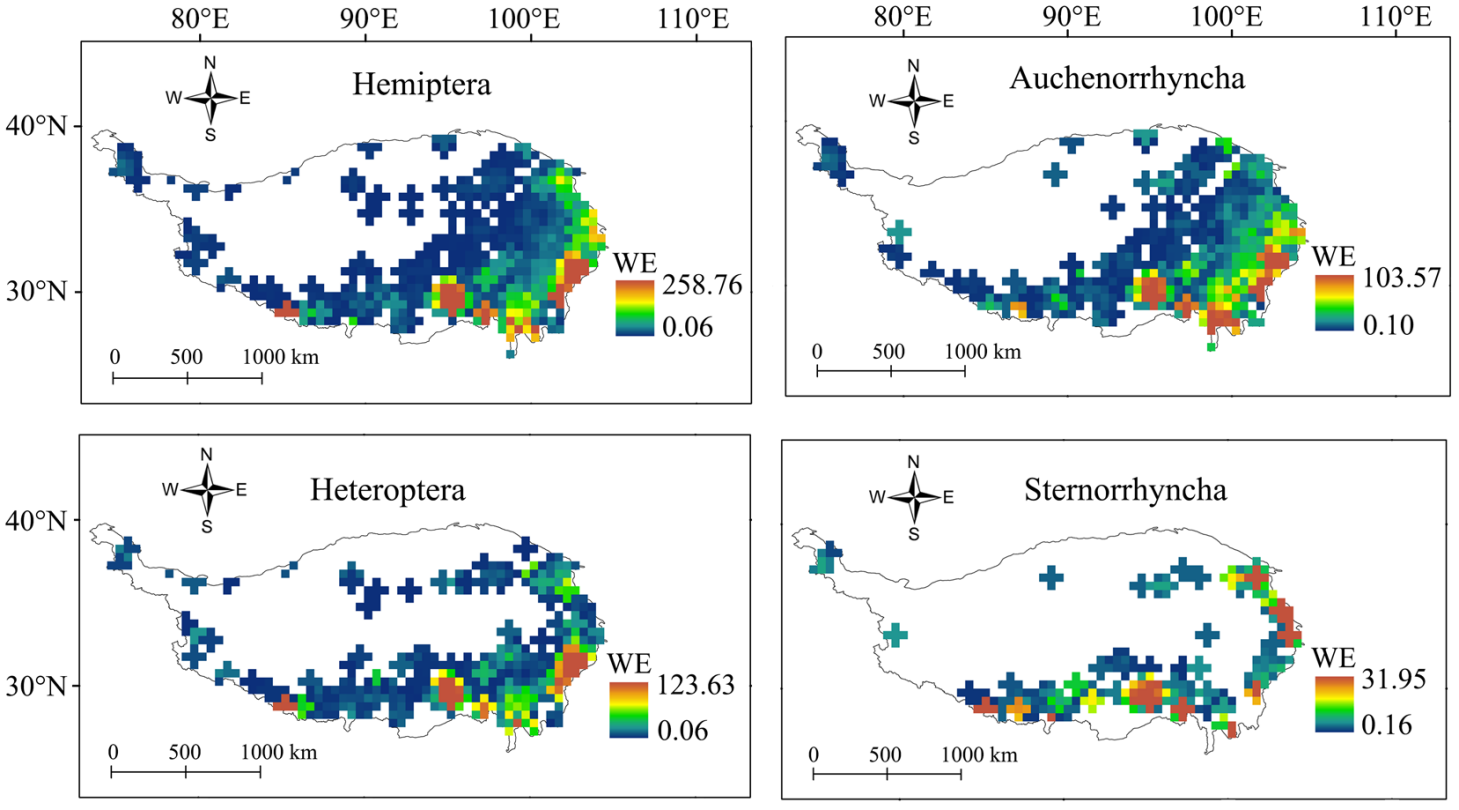
Weighted endemism (WE) patterns for Hemiptera (overall+three suborders) based on 0.5° × 0.5°grids on the Qinghai–Tibetan Plateau (QTP). Colors from dark blue to brown indicate higher WE values.

The WE values for Hemiptera on the QTP reflected heterogeneous distribution patterns (Figure 3). Values were high mainly in the southern and eastern regions and lower in the central and northern regions (Figure 3). The WE patterns for the three suborders were generally similar to that of Hemiptera as a whole; however, the distribution ranges differed (Figure 3). Auchenorrhyncha had the largest distribution range, followed by Heteroptera and Sternorrhyncha.

**FIGURE 4:**
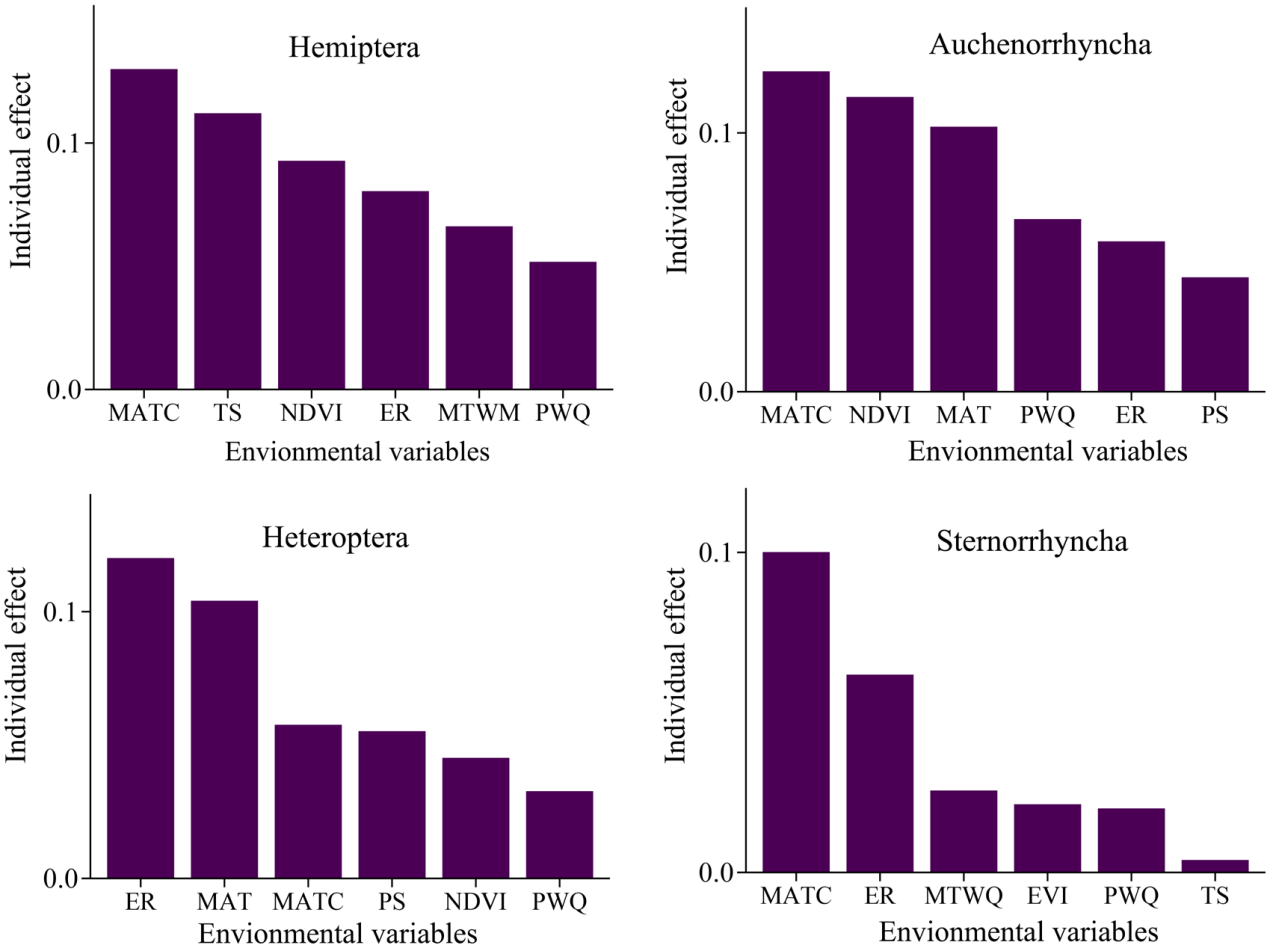
Independent effect of each environmental variable on Hemiptera WE (overall+three suborders) on the Qinghai‐Tibetan Plateau (QTP).

The univariate OLS model showed that climate seasonality (TS and PS) and historical climate change (MATC) were negatively correlated with the WE of Hemiptera (Table 1). No significant correlations were detected for TAR and APC, whereas the remaining variables were positively correlated with WE. The relationship between environmental variables and WE patterns of the three suborders was consistent with that observed for Hemiptera (Table 1), with the non‐significant effect of PS on Sternorrhyncha being the only exception. Moreover, the results of hierarchical partitioning indicated that all six categories of environmental variables determined the WE patterns of Hemiptera on the QTP, although their relative importance varied (Figure 4). MATC, which represented historical climate change, was the most critical environmental variable, followed by TS (climate seasonality) and NDVI (vegetation), in this order. ER (topographic heterogeneity), MTWM (temperature), and PWQ (precipitation) were less important. These results further demonstrated that the key determinants of endemism patterns varied across suborders (Figure 4). Specifically, the most important environmental variables shaping the endemism patterns of Auchenorrhyncha and Sternorrhyncha were MATC (historical climate change) (Figure 4). In contrast, ER (topographic heterogeneity) was a leading role for endemism patterns of Heteroptera (Figure 4).

**TABLE 1:** Results of the univariate ordinary least squares (OLS) model based on environmental variables and weighted endemism (WE) values.

Environmental variables	Hemiptera		Auchenorrhyncha		Heteroptera		Sternorrhyncha	
	Coef	*R* ^2^	Coef	*R* ^2^	Coef	*R* ^2^	Coef	*R* ^2^
MAT	0.59***	0.34	0.58***	0.30	0.51***	0.27	0.34***	0.09
MTWM	0.30***	0.08	0.27**	0.06	0.28**	0.07	0.13*	0.01
MTWQ	0.43***	0.18	0.43***	0.16	0.39***	0.15	0.24**	0.06
AP	0.52**	0.26	0.65**	0.34	0.45**	0.22	0.27***	0.07
PWQ	0.44*	0.19	0.56**	0.25	0.38*	0.15	0.23**	0.06
TAR	−0.07 ns	0.00	−0.12 ns	0.01	−0.06 ns	0.00	0.05 ns	0.00
TS	−0.52*	0.26	−0.54*	0.27	−0.46**	0.21	−0.15*	0.02
PS	−0.32**	0.09	−0.42**	0.10	−0.38***	0.15	−0.04 ns	0.00
ER	0.45***	0.20	0.40**	0.17	0.51***	0.25	0.37***	0.12
SP	0.49**	0.23	0.45**	0.22	0.47***	0.23	0.27***	0.07
TRI	0.49**	0.24	0.46**	0.22	0.46***	0.22	0.26***	0.07
EVI	0.47*	0.22	0.54**	0.23	0.39*	0.15	0.29**	0.07
NDVI	0.53*	0.27	0.63**	0.31	0.42*	0.18	0.29**	0.08
MATC	−0.57**	0.32	−0.65***	0.36	−0.47**	0.20	−0.39***	0.18
APC	−0.11 ns	0.01	−0.24 ns	0.04	−0.16 ns	0.02	−0.03 ns	0.00

*Note:* ***p < 0.001, **p < 0.01, *p < 0.05, ns, not significant (*p* > 0.05).

**FIGURE 5:**
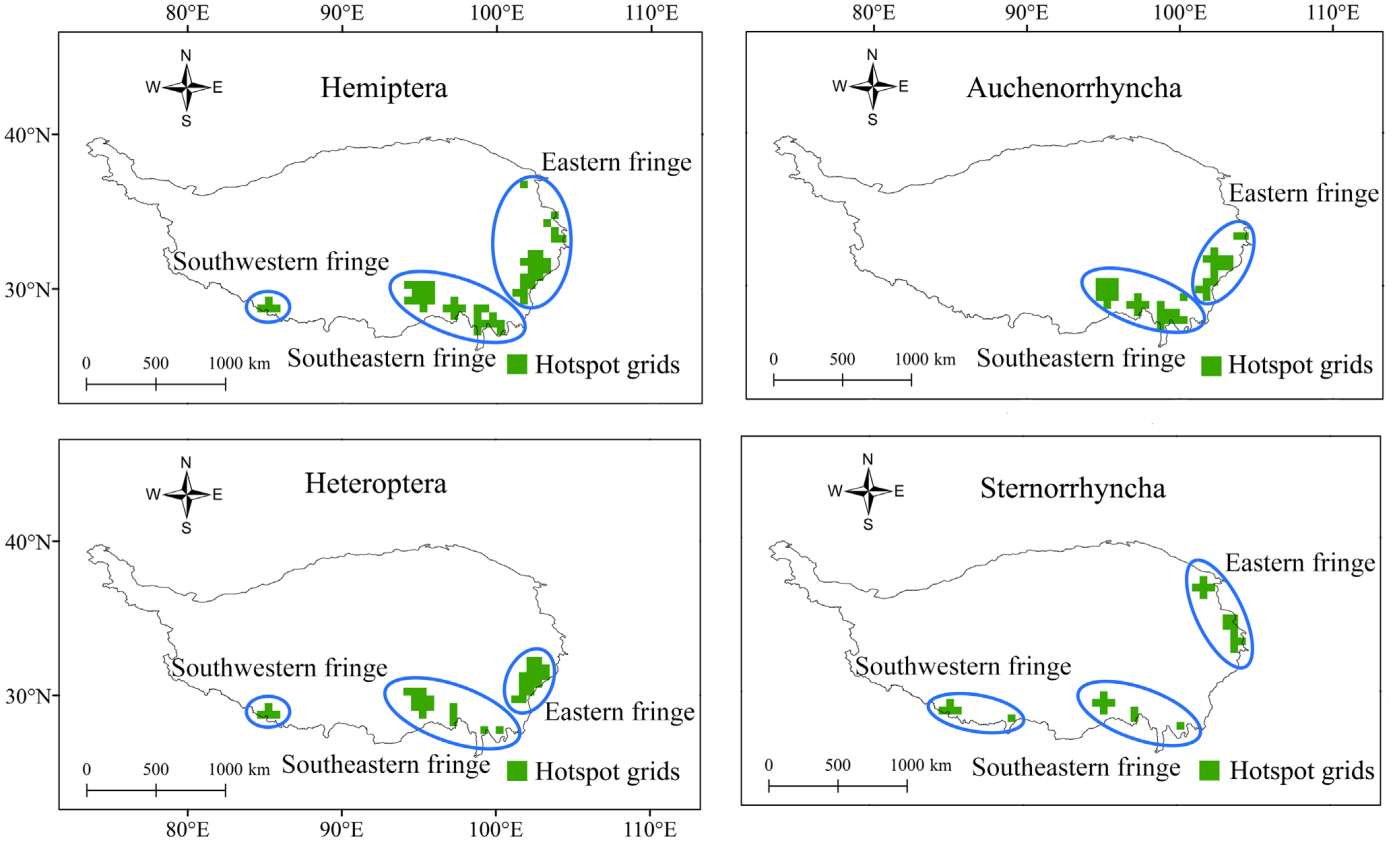
Top 10% of grids with the highest weighted endemism (WE) values identifying endemism hotspots for Hemiptera (overall+three suborders) on the Qinghai–Tibetan Plateau (QTP). Endemism hotspot grids are represented in green, with blue ellipses defining endemism hotspot regions.

**FIGURE 6:**
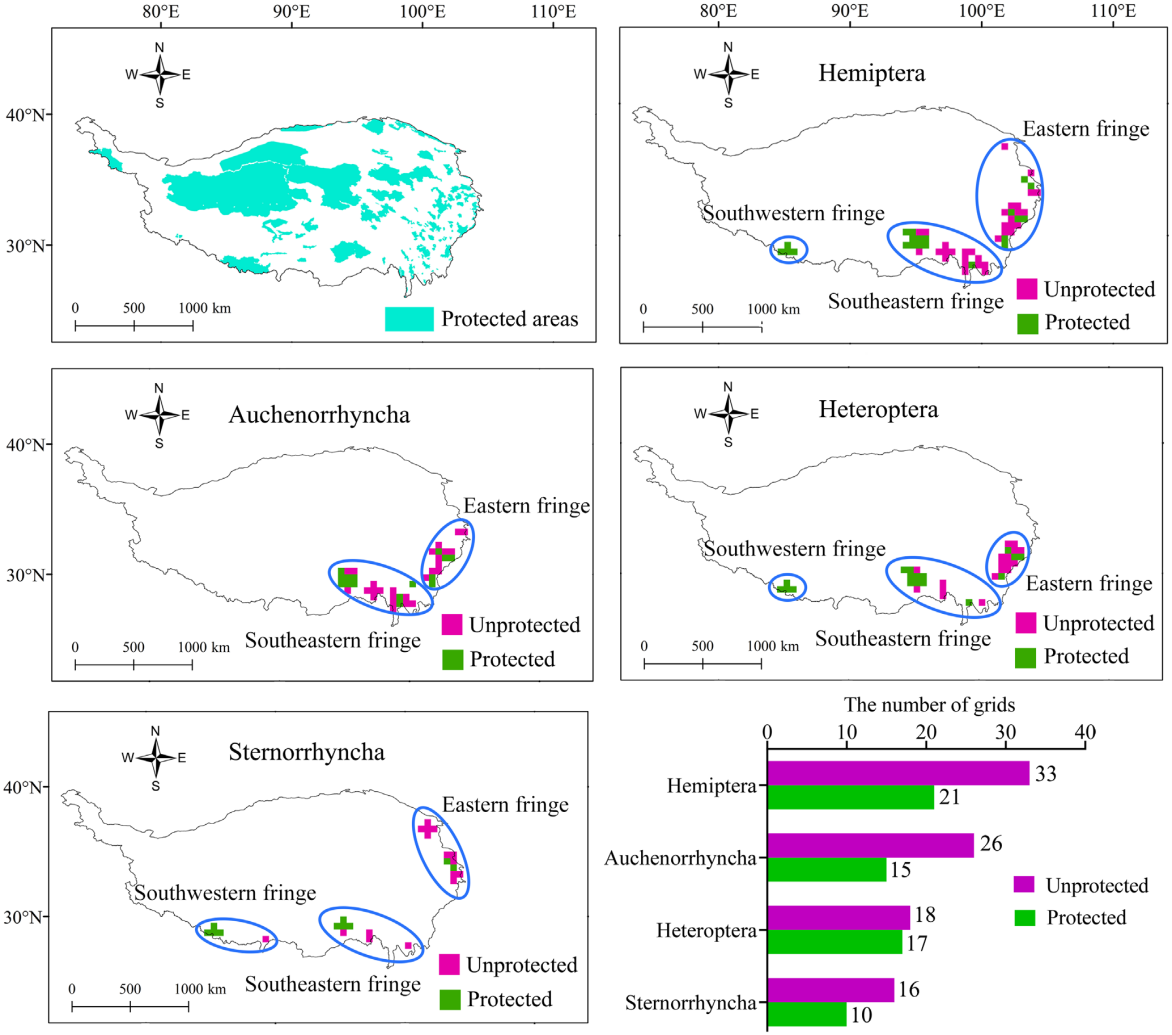
Conservation status of endemism hotspot grids for Hemiptera (overall+three suborders) on the Qinghai–Tibetan Plateau (QTP). The purple hotspot grids indicate the ineffective protection of Hemiptera in the protected areas, while the green hotspot grids represent that Hemiptera are safeguarded in the protected areas. Blue ellipses represent the defined endemism hotspot regions as shown in Figure 5.

A total of 54 hotspot grids for Hemiptera on the QTP were identified and clustered into three larger hotspots located in the eastern, southeastern, and southwestern fringes (Figure 5). A total of 41, 35, and 26 hotspot grids were obtained for Auchenorrhyncha, Heteroptera, and Sternorrhyncha, respectively. The hotspot grids for the latter two suborders were concentrated in the eastern, southeastern, and southwestern fringes, whereas those for Auchenorrhyncha were concentrated only in the eastern and southeastern fringes (Figure 5). Among the 54 hotspot grids for Hemiptera, 33 (61.11%) were not part of protected areas (Figure 6). When considering the three suborders, 26 of the 41 hotspot grids (63.41%) for Auchenorrhyncha, 18 of the 35 hotspot grids (51.42%) for Heteroptera, and 16 of the 26 hotspot grids (61.53%) for Sternorrhyncha were outside protected areas (Figure 6). Further analysis revealed that the unprotected hotspot grids for Hemiptera, Auchenorrhyncha, and Heteroptera were located in the southeastern and southwestern fringes (Figure 6), whereas those for Sternorrhyncha were located in all three fringes. Overall, the endemism hotspots for Hemiptera on the QTP are poorly safeguarded by protected areas.

## 4 Discussion

As we hypothesized, historical climate change has the strongest association with Hemiptera endemism patterns on the QTP. This result is in contrast with a previous conclusion regarding the key predictor of species richness patterns as reported in Li et al. (2019). Similar results were also found in other taxa and regions, such as Delphacidae and Gesneriaceae in China (Liu et al. 2017; Zhao et al. 2020) or plants in Europe and Mesoamerica (Svenning and Skov 2007; Sosa and Loera 2017). The results of this study and those of previous findings clearly emphasize that endemism patterns are primarily driven by unique mechanisms that differ from those shaping species richness patterns across taxa and regions. Thus, research on narrow‐ranging species with a relatively high level of endemism may fail to uncover important historical information about the mechanisms driving species diversity patterns if they ignore the endemism aspect. The most significant explanation for the strong association between historical climate change and endemism patterns is that species were often confined to climate refuges during glacial periods, and those with narrow distribution ranges were less mobile and less likely to leave these refuges after the end of a glacial period, even if suitable habitats were available elsewhere (Tribsch 2004; Svenning and Skov 2007; Zhao et al. 2020), which means they were significantly influenced by historical climate. This explanation is supported by the results of the present study, because three endemism hotspots for Hemiptera were identified in fringe regions of the QTP that are considered to not have been significantly affected by glaciations (Li et al. 2019).

Our study found that temperature seasonality is positively associated with endemism patterns, which suggests that smaller temperature variations promote the survival of narrow‐ranging species and contribute to high endemism. Additionally, our results revealed a positive correlation between vegetation and endemism. This finding may be related to the herbivorous nature of many Hemipteran species that have a close relationship with their host plants (Du et al. 2020). These host plants not only provide food but also offer diverse habitats (Blackman and Eastop 1994; Yuan et al. 2014; Zhao et al. 2021), both of which promote species survival. In contrast with our results, the previously mentioned study by Li et al. (2019) using a multivariate regression framework showed that topographic heterogeneity was the most important environmental variable determining species richness patterns. These results suggest that topographic heterogeneity primarily increases the available niche space and speciation. Temperature and precipitation have a relatively low influence on endemism patterns; however, temperature is more important, indicating that species prefer warmer environmental conditions.

The results of this study revealed that endemism patterns of Auchenorrhyncha and Sternorrhyncha are highly related to the same key environmental variables as those of Hemiptera, i.e., historical climate change (Figure 4). This may be due to the high total number of species in the two suborders (accounting for 61.41% of the total), which causes their response to environmental variables to predominantly drive the overall endemism patterns. In contrast, the endemism patterns of Heteroptera are mostly affected by topographic heterogeneity. These contrasting results reflect different mechanisms driving endemism patterns in different evolutionary branches of a particular taxonomic group. Therefore, to thoroughly understand the mechanisms underlying species diversity patterns, it is also necessary to pay attention to differentiation occurring within the evolutionary branches of a particular group.

Endemism patterns on the QTP are heterogeneous, and it is necessary to determine which areas require priority protection. The endemism hotspots for Hemiptera identified in the eastern, southeastern, and southwestern fringes of the QTP in this study are characterized by high levels of endemism and can be ranked as top priorities for conservation. Protected areas are the most effective tool for conserving biodiversity (Rodrigues and Cazalis 2020), yet their coverage of biodiversity hotspots is frequently low. Indeed, the eastern and southeastern fringes (61.11% of hotspot grids) identified in this study were shown not to be effectively covered by existing protected areas (Figure 6). This finding revealed the low efficiency of protected areas in preserving the high levels of endemism of Hemiptera on the QTP, which is consistent with the hypothesis proposed. Consequently, in regional conservation strategies, the eastern and southeastern fringes should have priority attention in the expansion of existing protected areas or the establishment of new protected areas. At the same time, it must be clearly recognized that the expansion or establishment of protected areas requires sufficient funds as a prerequisite. Therefore, the local government departments should increase financial input and formulate long‐term financial plans to ensure the sustainable implementation of conservation strategies.

The formation of species diversity patterns on the QTP is a complex process. One limitation of the present study is that it did not consider the significance of uplift, which is regarded as a key factor driving diversity (Xing and Ree 2017; Ye et al. 2019; Ding et al. 2020). Future research on Hemiptera on the QTP could integrate several methods, such as molecular dating and diversification rate analyses, to clarify whether uplift promotes the formation of endemism patterns through increased diversification. Additionally, incorporating phylogenetic endemism can provide insights into the evolutionary mechanisms underlying species diversity patterns; it can also assist in identifying priority conservation areas based on multiple dimensions of biodiversity as well as in further optimizing the protected area network.

## 5 Conclusion

The results of this study showed that endemism patterns on the QTP are primarily driven by historical climate change. Additionally, we identified three priority conservation areas (eastern, southeastern, and southwestern fringes) based on the detection of endemism hotspots and observed that current protected areas provide insufficient coverage for the eastern and southeastern fringes, indicating a significant conservation gap. Overall, this study highlights that endemism plays an indispensable role when trying to understand the mechanisms driving species diversity and that a more comprehensive network of protected areas is urgently needed.

## Author Contributions

**Zhengxue Zhao:** conceptualization (lead), data curation (lead), formal analysis (lead), funding acquisition (supporting), writing – original draft (lead). **Xueli Feng:** conceptualization (supporting), data curation (supporting). **Jing Zhou:** data curation (supporting). **Xiudong Huang:** data curation (supporting).

## Funding

This work was supported by Guizhou Provincial Science and Technology Projects (QKHJC[2024]youth290, QKHJCMS[2025]075) and Scientific Research Platform of Education Department of Guizhou Province (Qianjiaoji [2022]052).

## Conflicts of Interest

The authors declare no conflicts of interest.

## Supporting information


**Table S1:** Occurrence records of Hemiptera species on the QTP, including suborder, family, genus, species, and corresponding geographic coordinates.

## Data Availability

The occurrence records of Hemiptera on the QTP (Table S1) and the R codes are provided in supporting materials.
